# Supplementary material for: Temporospatial hierarchy and allele-specific expression of zygotic genome activation revealed by distant interspecific urochordate hybrids
Source: Nat Commun. 2024 Mar 16;15:2395. doi: 10.1038/s41467-024-46780-0 (PMC10944513; doi:10.1038/s41467-024-46780-0)
Supplement: Supplementary file 1 — Supplementary Information [file 41467_2024_46780_MOESM1_ESM.pdf]

Supplementary figures

Temporospatial hierarchy and allele-specific expression of zygotic genome activation revealed by distant interspecific urochordate hybrids

Jiankai Wei[1,2,3#], Wei Zhang[1#], An Jiang[1#], Hongzhe Peng[1#], Quanyong Zhang[4#], Yuting Li[1], Jianqing Bi[1], Linting Wang[5], Penghui Liu[1], Jing Wang[1], Yonghang Ge[1], Liya Zhang[4], Haiyan Yu[1], Lei Li[5], Shi Wang[1,2], Liang Leng[6*], Kai Chen[4,7*], Bo Dong[1,2,3*]

1. Fang Zongxi Center for Marine EvoDevo, MoE Key Laboratory of Marine Genetics and Breeding, College of Marine Life Sciences, Ocean University of China, Qingdao 266003, China;
2. Laboratory for Marine Biology and Biotechnology, Qingdao Marine Science and Technology Center, Qingdao 266237, China;
3. MoE Key Laboratory of Evolution and Marine Biodiversity, Institute of Evolution and Marine Biodiversity, Ocean University of China, Qingdao 266003, China;
4. State Key Laboratory of Primate Biomedical Research and Institute of Primate Translational Medicine, Kunming University of Science and Technology, Kunming, Yunnan 650500, China;
5. National Center of Mathematics and Interdisciplinary Sciences, Academy of Mathematics and Systems Science, Chinese Academy of Sciences, Beijing, 100190, China;
6. Institute of Herbgenomics, Chengdu University of Traditional Chinese Medicine, Chengdu 611137, China.
7. Current address: Southern Marine Science and Engineering Guangdong Laboratory (Guangzhou), No. 1119 Haibin Rd, Nansha Dist., Guangzhou 511458, China.

# These authors contribute to this article equally.

*Correspondence, bodong@ouc.edu.cn (B.D.); chen_kai@gmlab.ac.cn (K.C.); lleng@icmm.ac.cn (L.L.)

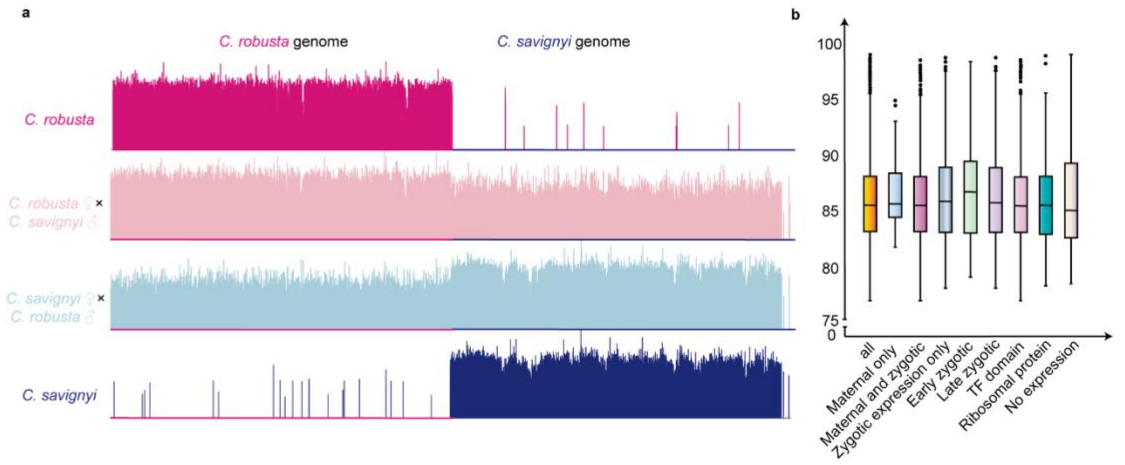

Supplementary Fig. S1 | The gene sequences between *C. robusta* and *C. savignyi* are divergent sufficient to be distinguished. a, The statistics of reads mapping to the genome of both *C. robusta* and *C. savignyi* at late neurula stage. The horizontal axis represents genome position and the vertical axis represents the log2 transformed reads number in each window. The left part represented genome of *C. robusta*, the right part represented the genome of *C. savignyi*. Four ascidians included self-crossing *C. robusta*, *C. savignyi* as male parent cross with *C. robusta* (Cr♀ × Cs♂), *C. robusta* as male parent cross with *C. savignyi* (Cs♀ × Cr♂), and self-crossing *C. savignyi*. **b,** The identity of orthologous genes between *C. robusta* and *C. savignyi*. The horizontal axis represents different gene sets and the vertical axis represents the identity level between orthologous genes.

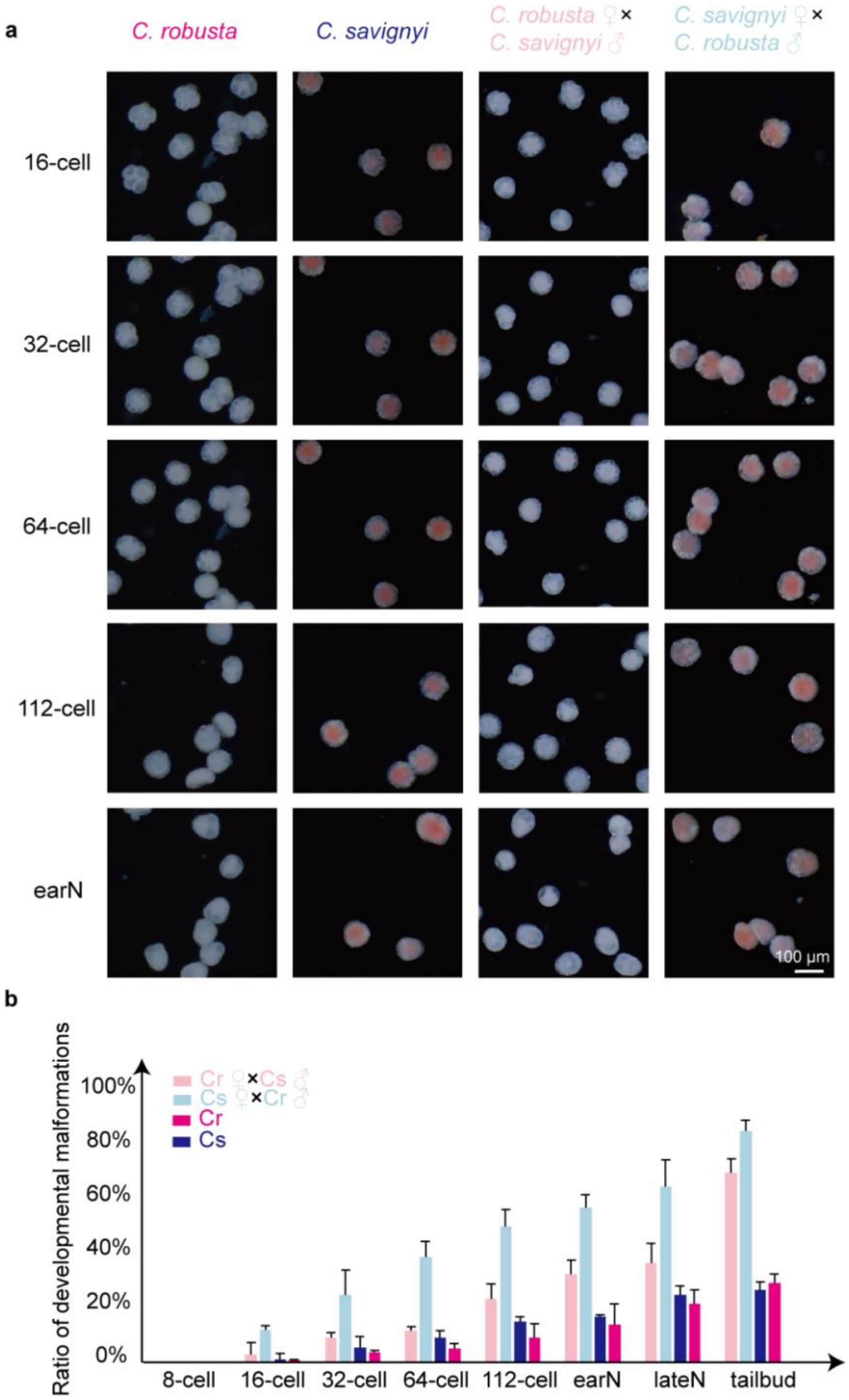

Supplementary Fig. S2 The morphological observation and the statistics of the developmental failure of *C. robusta*, *C. savignyi*, and hybrid animals. a, The morphology of five developmental stages were observed including 16-cell, 32-cell, 64-cell, 112-cell, and early-neurula (earN) stage. Scale bar: 100 μm. **b,** The statistics of developmental failure rates from 8-cell stage. The abnormal embryos were regarded as the developmental failure embryos.

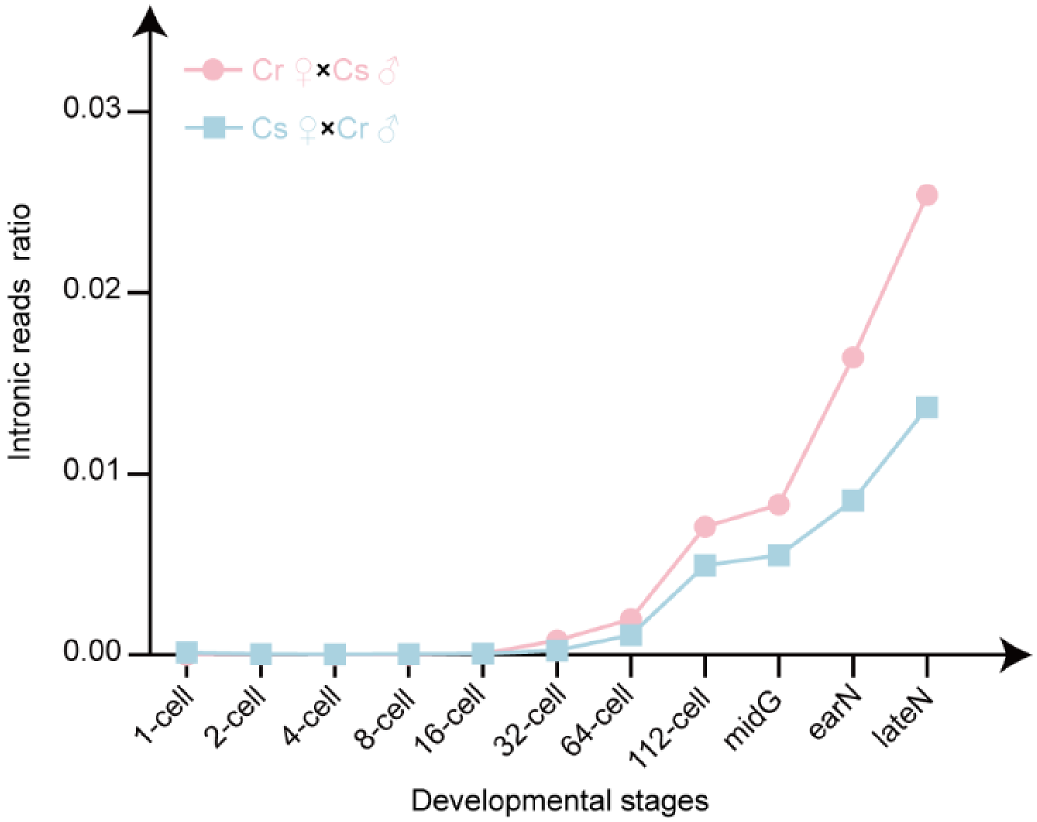

Supplementary Fig. S3 | The statistics of intronic reads ratio to total reads at different developmental stages. The different developmental stages are listed at the horizontal axis and the intronic read ratio to all reads are listed at the vertical axis for Cs♀ × Cr♂ samples and Cr♀ × Cs♂ samples.

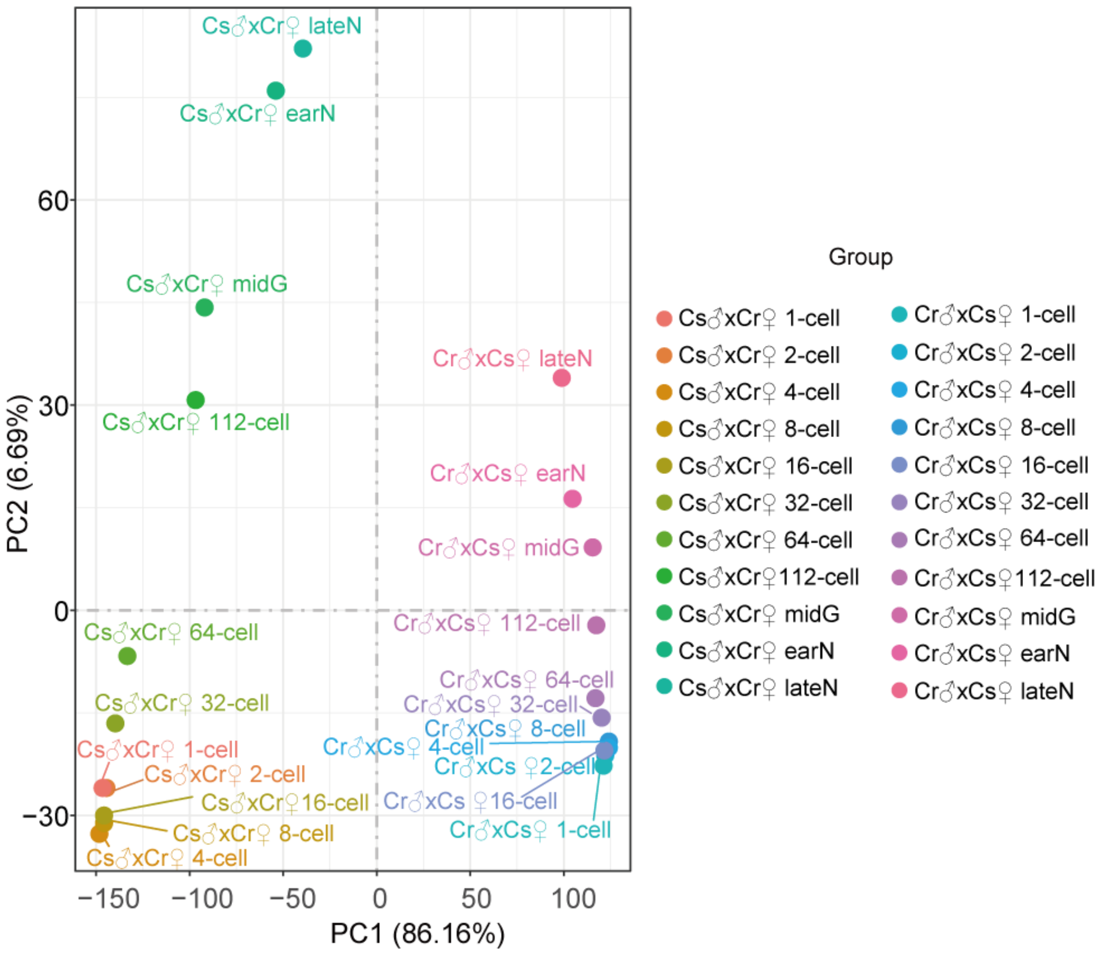

Supplementary Fig. S4 | The principal component analysis of bulk RNA-seq samples according to gene FPKM value. PC1 divided the samples into two parts, Cs♀ × Cr♂ samples and Cr♀ × Cs♂ samples, which corresponded with their parents. PC2 divided the samples into two parts. Samples from 1-cell to 64-cell stage were clustered into one group, indicating that their expression was featured with maternal-derived mRNAs. Samples from mid-gastrula to late-neurula stage were clustered into another group, indicating that their expressions were featured with zygotic-transcribed mRNAs. Different colors represent different samples.

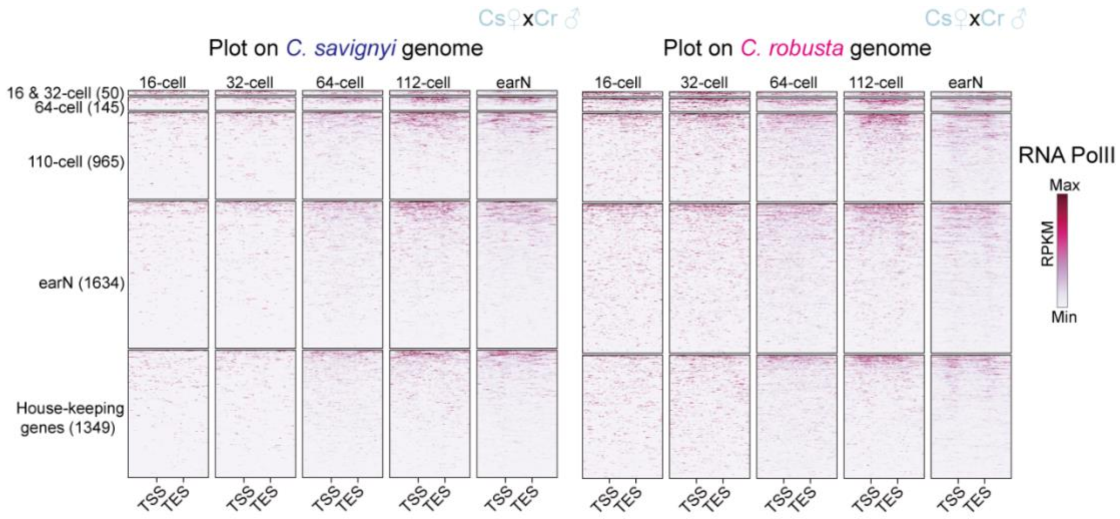

Supplementary Fig. S5 | The occupancy of RNA pol II indicated both maternal and paternal genomic activation. Heat map of Pol II CUT&Tag signals across activated genes (FPKM of RNA-seq data larger than 1, grouped by stages in rows) and housekeeping genes in Cs♀ × Cr♂ hybrid embryos. Each line showed the normalized signals for a gene (homolog gene pair from *C. savignyi* or *C. robusta*) from -2k bp above TSS to +2k bp below TES. More than two biological replicons with similar results of all developmental stages were produced by Pol II CUT&Tag experiments.

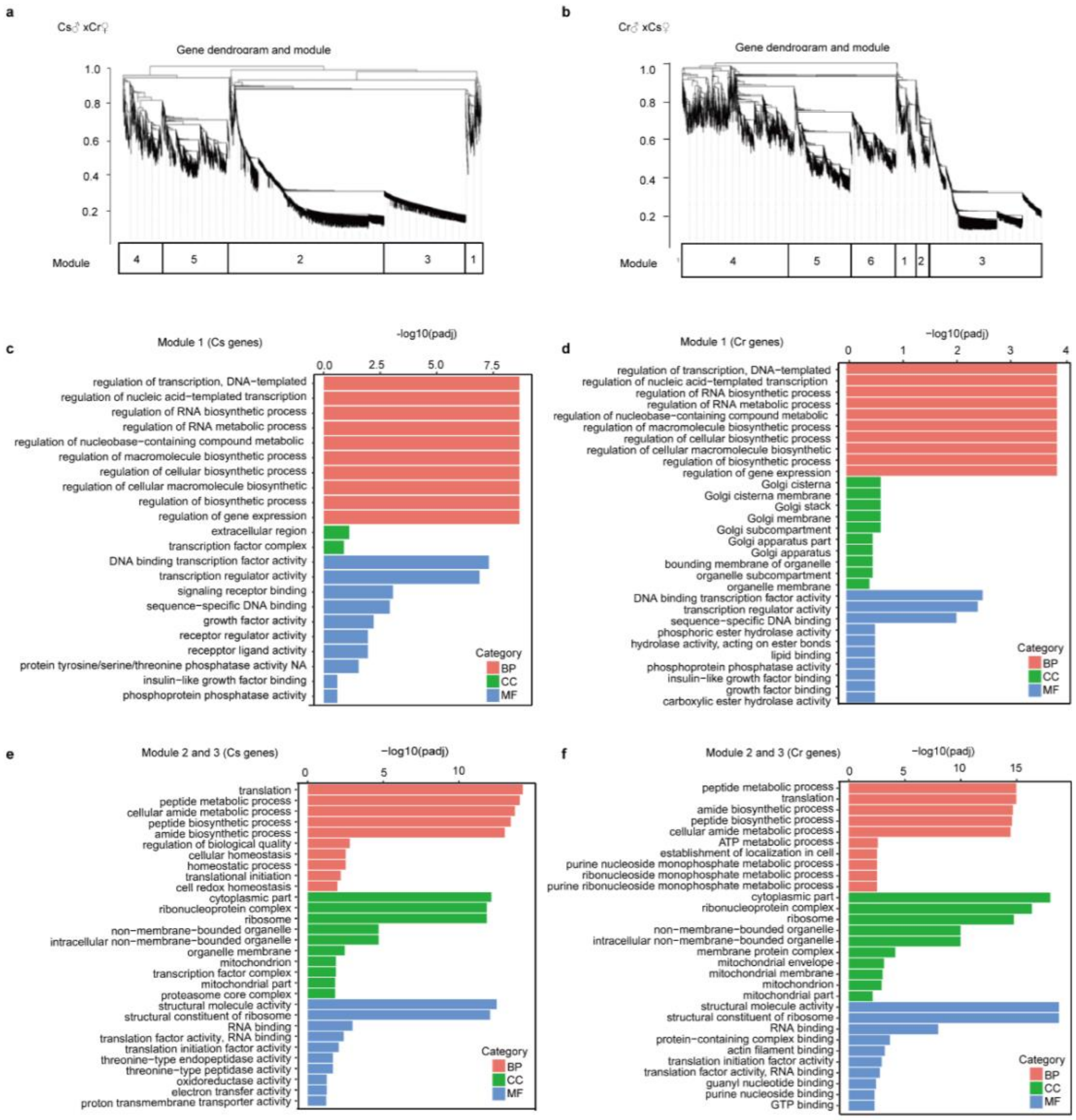

Supplementary Fig. S6 | WGCNA analysis of genes in hybrid embryos and GO enrichment analysis of each module. a, Hierarchical clustering tree showed that genes were divided into 5 modules in Cr♀ × Cs♂ hybrid. **b**, Hierarchical clustering tree showed that genes were divided into 6 modules in Cs♀ × Cr♂ hybrid. **c**, GO enrichment analysis of paternal genes in module 1 in Cr♀ × Cs♂ hybrid. **d**, GO enrichment analysis of paternal genes in module 1 in Cs♀ × Cr♂ hybrid. **e**, GO enrichment analysis of paternal genes in module 2 and 3 in Cr♀ × Cs♂ hybrid. **f**, GO enrichment analysis of paternal genes in module 2 and 3 in Cs♀ × Cr♂ hybrid.

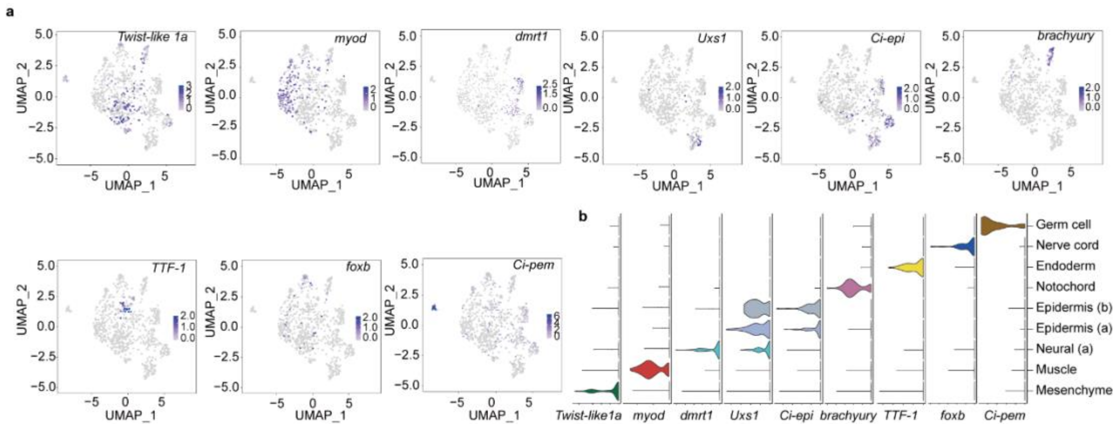

Supplementary Fig. S7 | Marker genes in UMAP and Vlnplot at 64-cell stage from Cr♀ × Cs♂ hybrid. a, UMAP projection and expression patterns of representative marker genes at 64-cell stage of hybrid embryos. *Twist-like1* gene in the cluster mesenchyme (n = 57 cells). *Myod* gene in the cluster muscle (n = 61 cells). *Dmrt1* gene in the cluster neural (n = 22 cells). *Uxs1* gene in the cluster epidermis (b) (n = 36 cells). *Ci-epi* gene in the cluster epidermis (a) (n = 39 cells). *Brachyury* gene in the notochord (n = 28 cells). *TTF-1* gene in the cluster endoderm (n = 20 cells). *Foxb* gene in the cluster nerve cord (n = 11 cells). *Ci-pem* gene in the cluster germ line (n = 8 cells). **b,** The marker gene expression level in each cell type. Gene names are listed at the horizontal axis, and cell clusters are listed at the vertical axis.

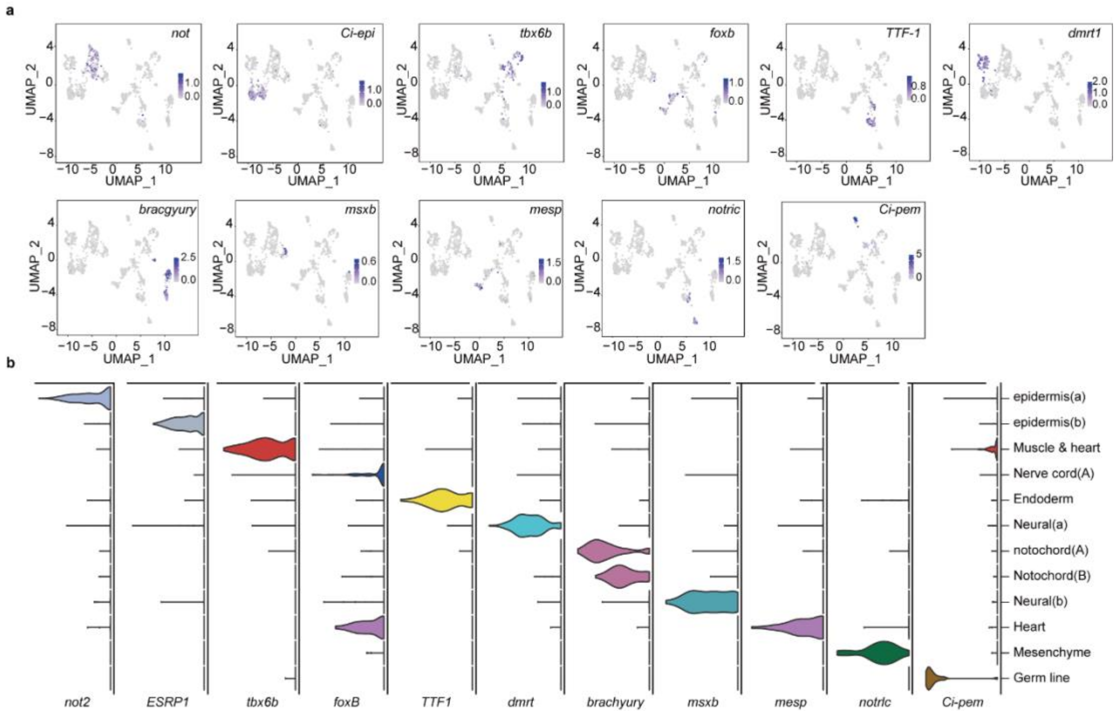

Supplementary Fig. S8 | Marker genes in UMAP and Vlnplot at 112-cell stage from Cr♀ × Cs♂ hybrid. a, UMAP projection and expression patterns of representative marker genes at 112-cell stage of hybrid embryos. *Not* gene in the cluster Epidermis (a) (n = 87 cells). *Ci-epi* gene in the cluster Epidermis (b) (n = 73 cells). *Tbx6b* gene in the cluster muscle (n = 54 cells). *Foxb* gene in the cluster nerve cord (A) (n = 37 cells). *TTF-1* gene in the cluster endoderm (A) (n = 66 cells). *Dmrt* gene in the neural (a) (n = 76 cells). *Brachyury* gene in two clusters notochord (n = 114 cells). *Msxb* gene mapped in the cluster neural (b) (n = 15 cells). *Mesp* gene in the cluster heart (n = 15 cells). *Notrlc* gene in the cluster mesenchyme (n = 22 cells). *Ci-pem* gene in the cluster germ line (n = 20 cells). **b,** The marker gene expression level in each cell type. Gene names are listed at the horizontal axis, and cell clusters are listed at the vertical axis.

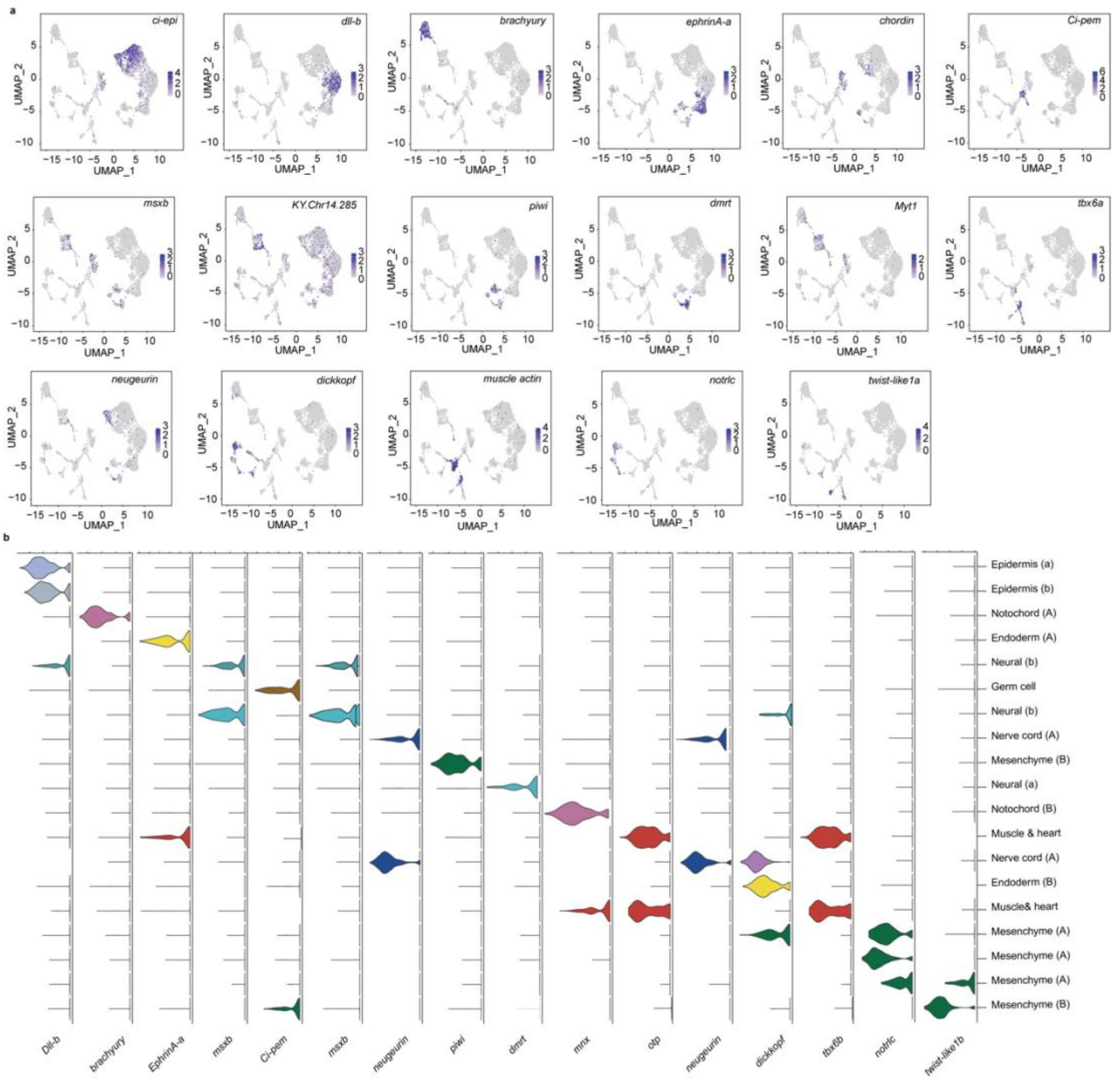

Supplementary Fig. S9 | Marker genes in UMAP and Vlnplot at early neurula stage from Cr♀ × Cs♂ hybrid. a, UMAP projection and expression patterns of representative marker genes of early neurula stage of hybrid embryos. *Brachyury* gene in the cluster notochord. *EphrinA* gene in the cluster endoderm. *Chordin* and *msxb* gene in the clusters neural (b), *Ci-pem* gene in the cluster germ line. *KY. chr14.285* gene in the clusters nerve cord. *Piwi*, *dmrt*, *myt1*, muscle *actin* and *tbx6a* genes in the cluster muscle/heart. *Neugeurin* gene in the cluster nerve cord. *Dickkopf* gene in the cluster endoderm (A), *piwi*, *notrlc*, and *twist-like1* genes in the cluster mesenchyme. **b,** The marker gene expression level in each cell type. Gene names are listed at the horizontal axis, and cell clusters are listed at the vertical axis.

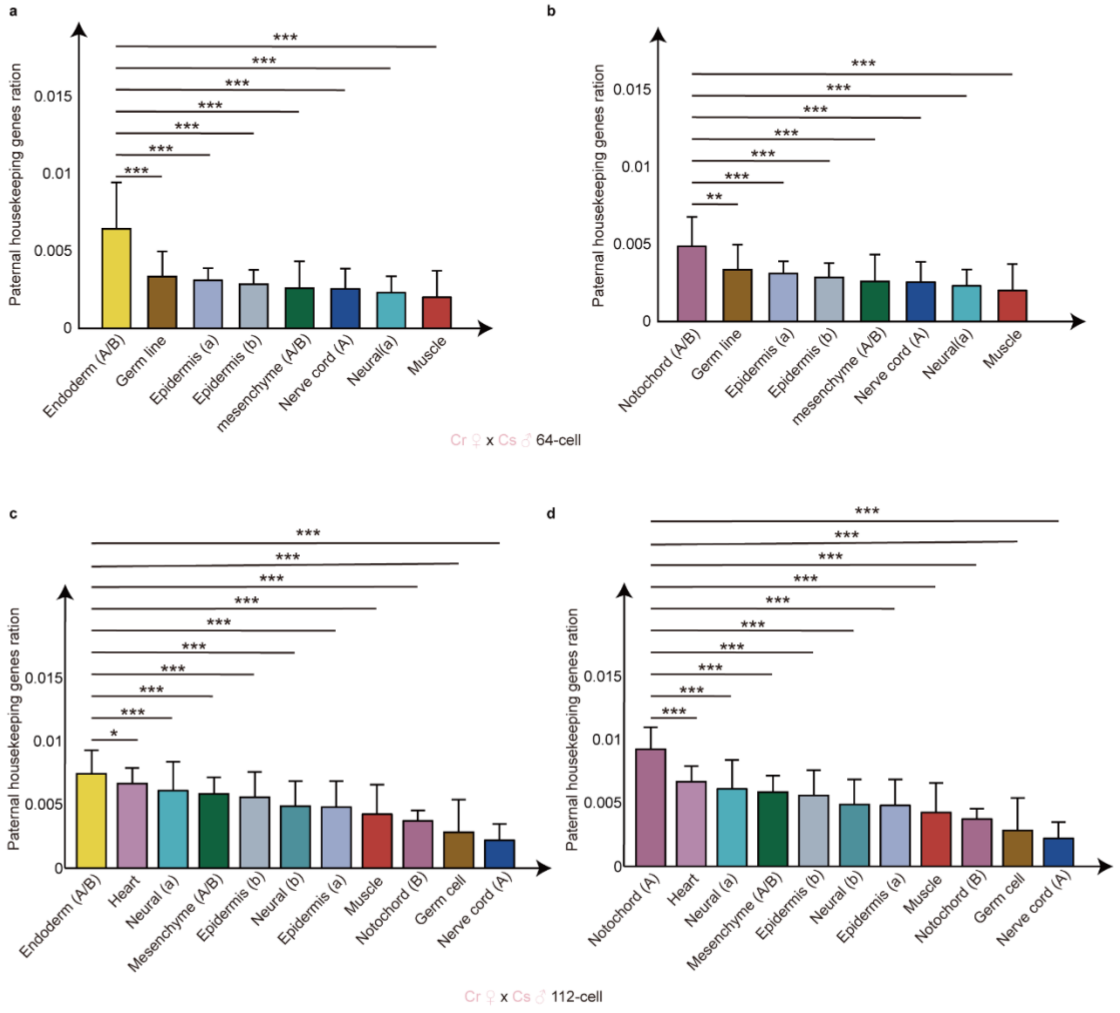

Supplementary Fig. S10 | The statistics of paternal housekeeping gene ratio in different cell clusters from Cr♀ × Cs♂ hybrid. **a,** Differential analysis of count ratio of paternal housekeeping genes in endoderm cells compared to other cell types at 64-cell stage. **b**, Differential analysis of count ratio of paternal housekeeping genes in notochord cells compared to other cell types at 64-cell stage. **c,** Differential analysis of count ratio of paternal housekeeping genes in endoderm cells compared to other cell types at 112-cell stage. **d**, Differential analysis of count ratio of paternal housekeeping genes in notochord cells compared to other cell types at 112-cell stage. *P*-value ≤ 0.05 was marked one asterisk as significant difference. *P*-value ≤ 0.001 was marked three asterisks as extremely significant difference.

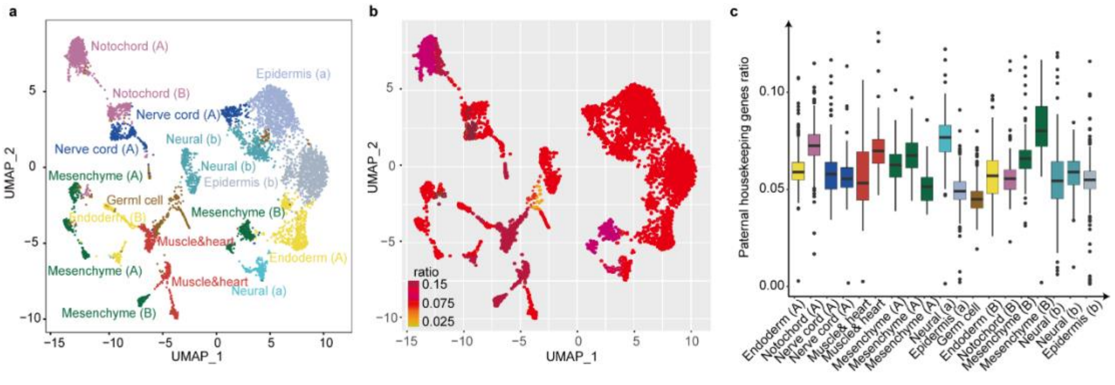

Supplementary Fig. S11 | Housekeeping gene activation revealed by single-cell transcriptomics at early neurula stage from Cr♀ × Cs♂ hybrid. a, The UMAP plot of the early neurula stage samples. 6,511 cells were divided into 19 clusters and annotated as nine cell types. The color font was corresponded to the cell types. **b**, The expression ratio of paternal housekeeping genes in each cell at early neurula stage. The color represented the expression ratio. **c**, The boxplot of expression ratio of paternal housekeeping genes in each cell types at early neurula stage. n = 517, 573, 260, 198, 209, 189, 136, 114, 87, 227, 1199, 348, 191, 221, 253, 83, 317, 296, 1094 cells for each cell type from endoderm to epidermis (b). In each box, the horizontal black lines represent median values; boxes extend from 25th to 75th percentile of each group's distribution of values; the vertical extending lines indicate adjacent values, and dots mark observations outside the range of adjacent values.

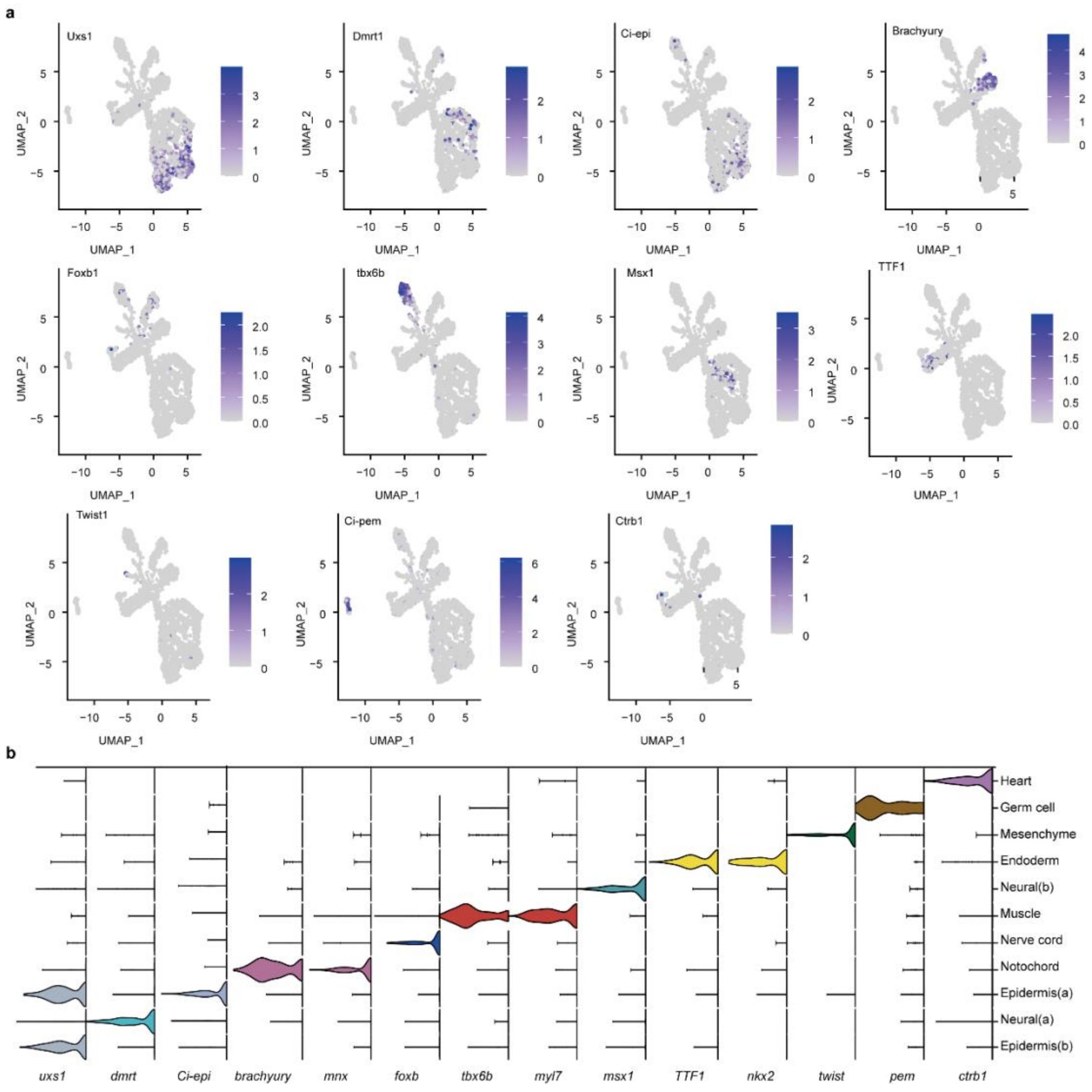

Supplementary Fig. S12 | Marker genes in UMAP and Vlnplot at 112-cell stage of Cs♀ × Cr♂ hybrid. a, UMAP projection and expression patterns of representative marker genes at 112-cell stage of hybrid embryos. **b,** The marker gene expression level in each cell type. Gene names are listed at the horizontal axis, and cell clusters are listed at the vertical axis.

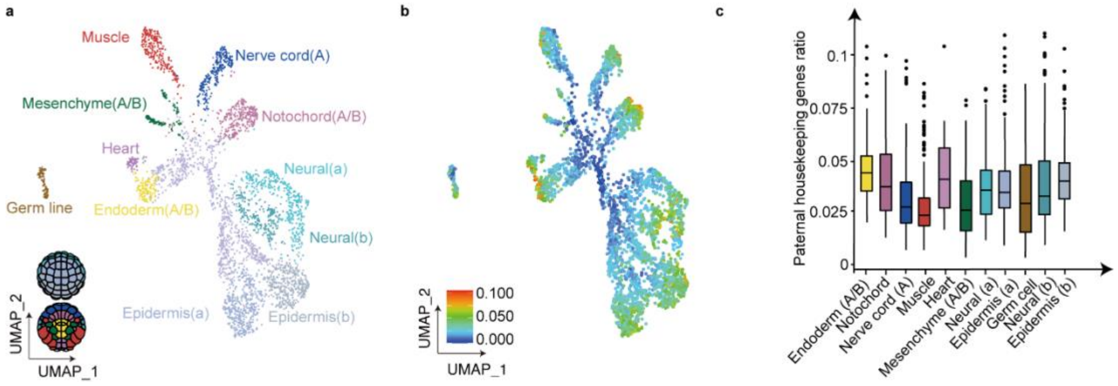

Supplementary Fig. S13 | Housekeeping gene activation revealed by single-cell transcriptomics from Cs♀ × Cr♂ hybrid. a, The UMAP plot of the 112-cell stage samples. The color font was corresponded to the cell types. **b**, The expression ratio of paternal housekeeping genes in each cell at 112-cell stage. The color represented the expression ratio. **c**, The boxplot of expression ratio of paternal housekeeping genes in each cell types at 112-cell stage. n = 135, 269, 245, 238, 63, 123, 393, 465, 91, 231, 380 cells for each cell type from endoderm to epidermis (b). In each box, the horizontal black lines represent median values; boxes extend from 25th to 75th percentile of each group's distribution of values; the vertical extending lines indicate adjacent values, and dots mark observations outside the range of adjacent values.

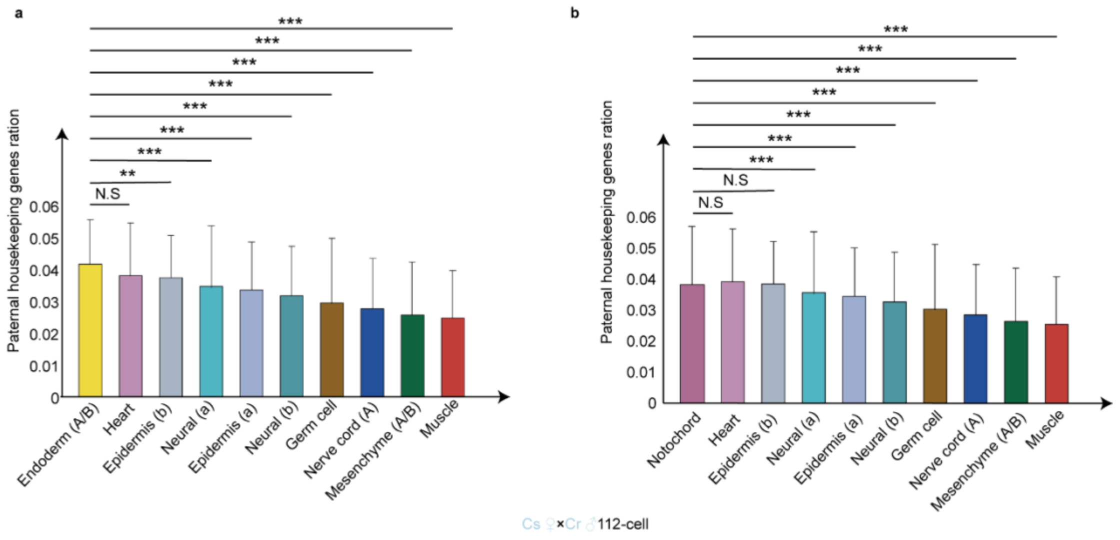

Supplementary Fig. S14 | The statistics of paternal housekeeping gene ratio in different cell clusters from Cs♀ × Cr♂ hybrid. **a,** Differential analysis of count ratio of paternal housekeeping genes in endoderm cells compared to other cell types at 112-cell stage. **b**, Differential analysis of count ratio of paternal housekeeping genes in notochord cells compared to other cell types at 112-cell stage. *P*-value ≤ 0.05 was marked one asterisk as significant difference. *P*-value ≤ 0.001 was marked three asterisks as extremely significant difference.

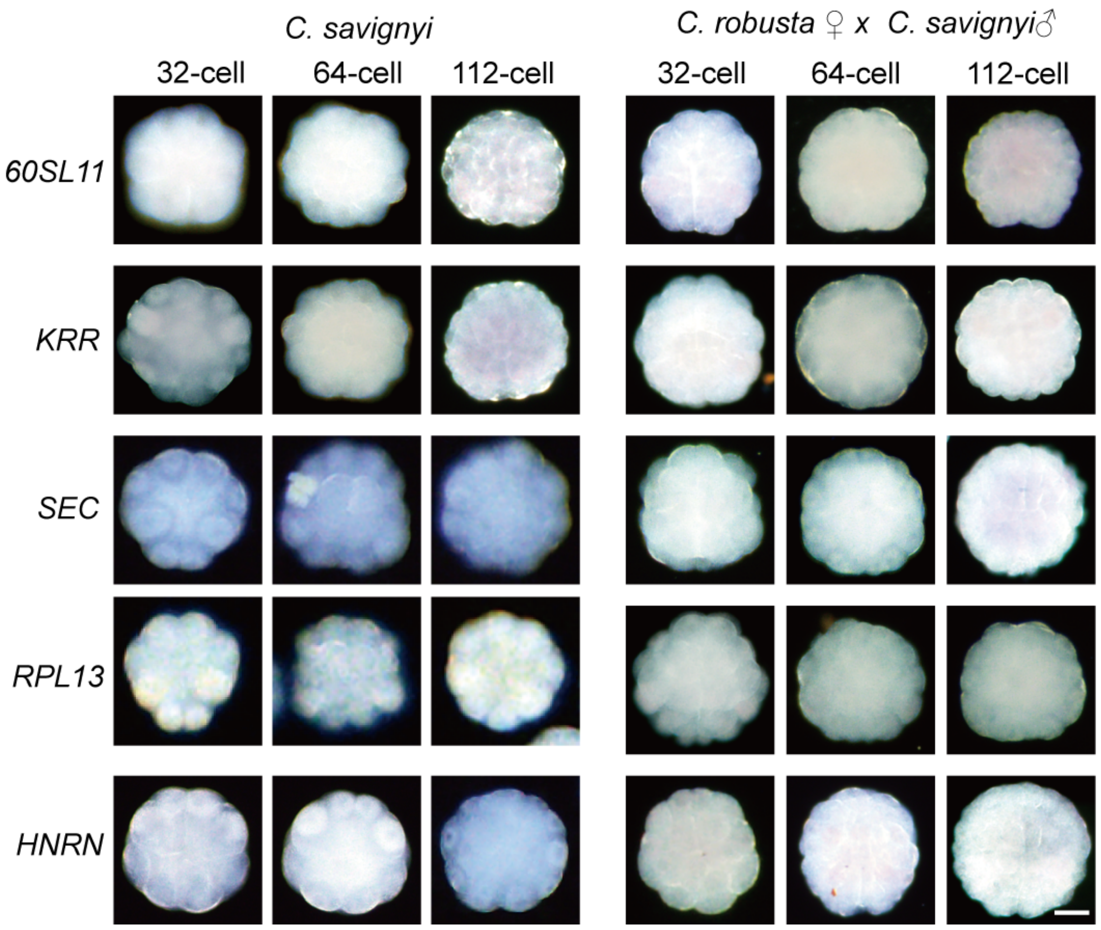

Supplementary Fig. S15 | Whole-mount *in situ* hybridization result of sense probes of *60SL11*, *KRR*, *SEC*, *RPL13*, and *HNRN*. The sense probes were applied to detect the signals in both self-cross *C. savignyi* embryos and Cr♀ × Cs♂ embryos at 32-cell, 64-cell, and 112-cell stages as control groups. No signals were detected for all the sense probes. Scale bar: 50 µm.

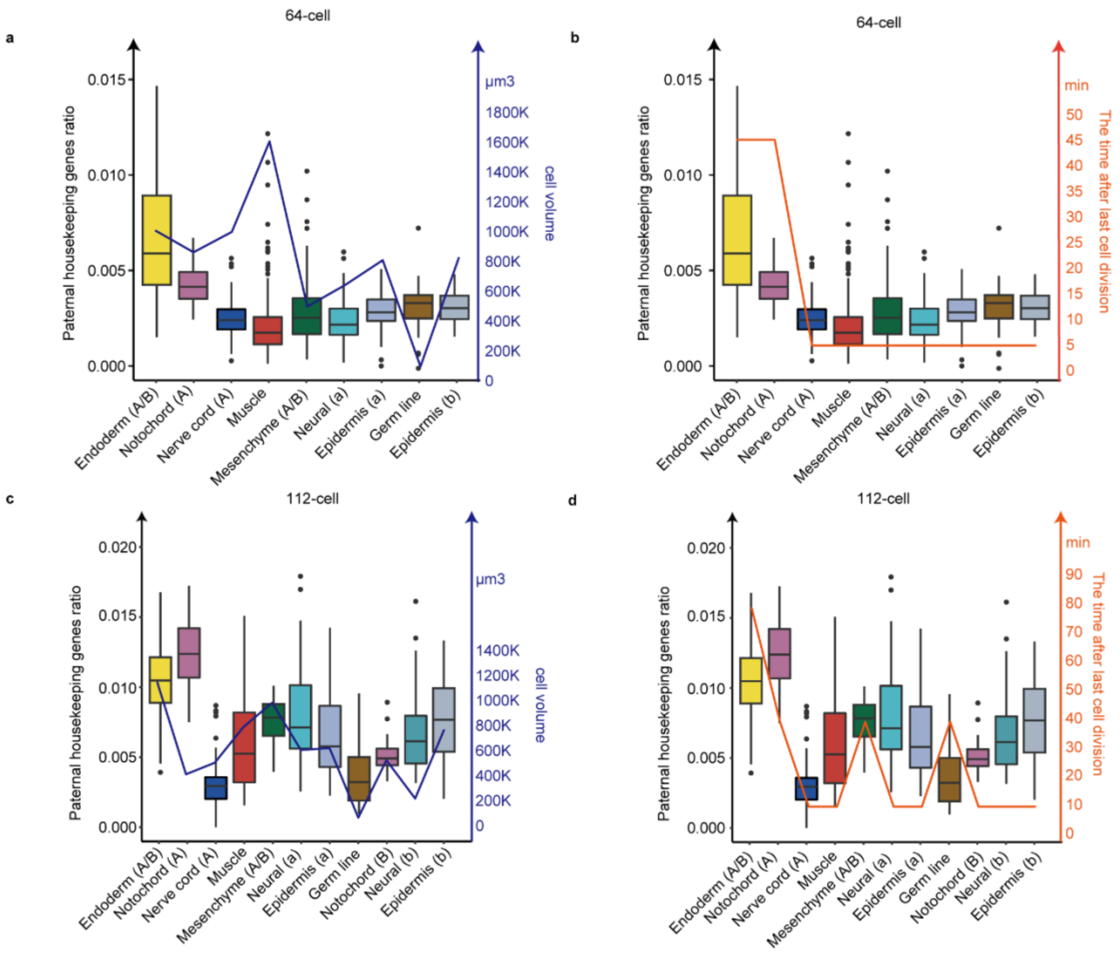

Supplementary Fig. S16 | The cell size and time after the last cell division in each cell type in *C. robusta*. a, Cell size and ratio of paternal housekeeping genes in each cell type at the 64-cell stage. **b,** Time after the last cell division and ratio of paternal housekeeping genes in each cell type at the 64-cell stage. **c,** Cell size and ratio of paternal housekeeping genes in each cell type at the 112-cell stage. **d,** Time after the last cell division and ratio of paternal housekeeping genes in each cell type at the 112-cell stage. Blue and orange represent cell volume and the time after the last time cell division, respectively.

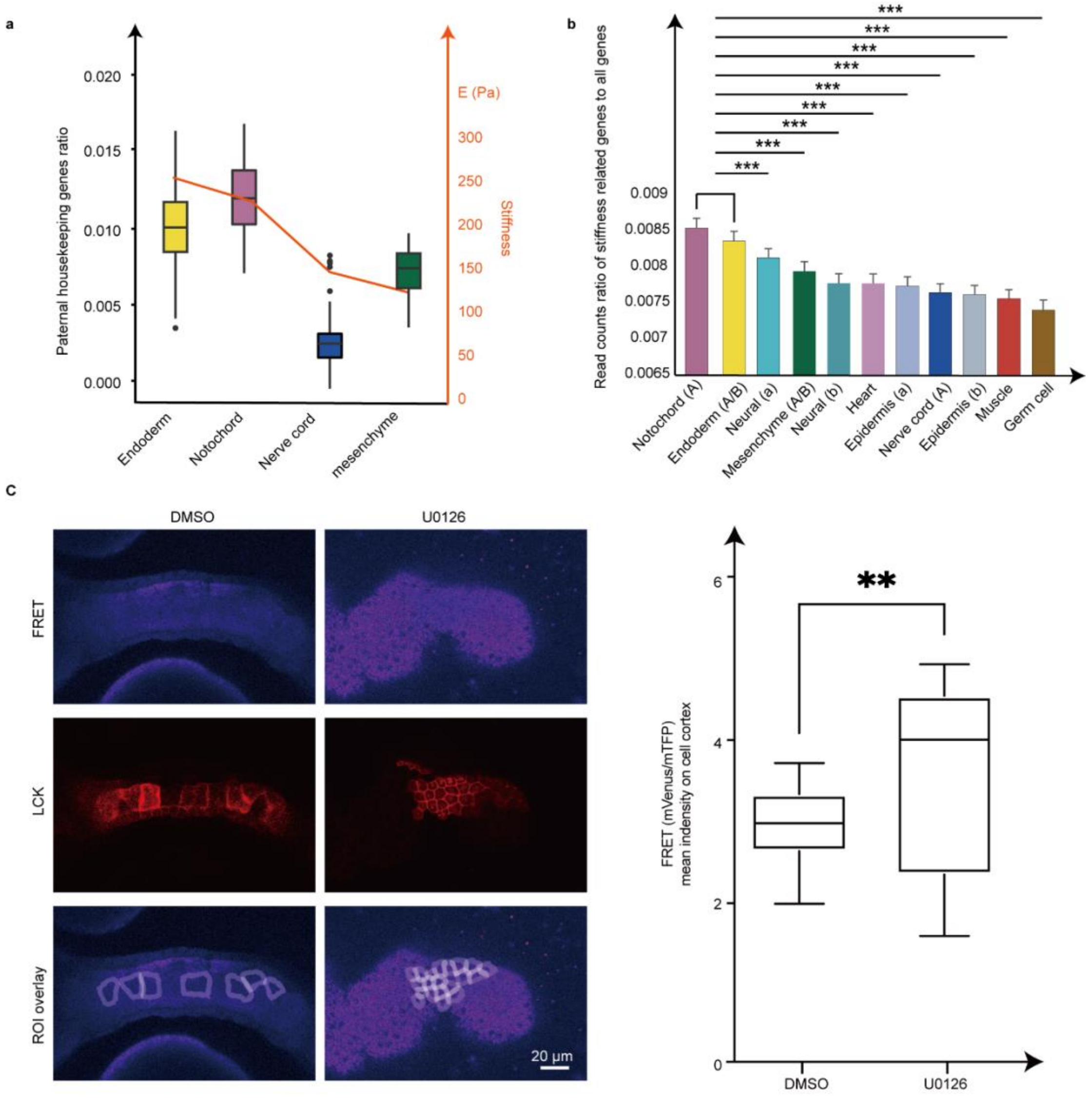

Supplementary Fig. S17 | The statistics of cell stiffness and stiffness-related genes according to count ratio in different cell clusters. a, Cell stiffness at 76-cell stage and boxplot of the expression ratio of paternal housekeeping genes in different cell types at the 112-cell stage. Orange line represents the stiffness values. **b,** Differential analysis of read counts ratio of stiffness related genes in endoderm and notochord cells compared to other cell types. p value ≤ 0.001 was marked three asterisks as extremely significant difference. **c,** Cell stiffness measurement after cell fate transfer. U0126 was used to transfer the cell fate of notochord. The stiffness was measured with a vinculin tension sensor based on FRET at the mid-tailbud stage. Scale bar: 20 μm.

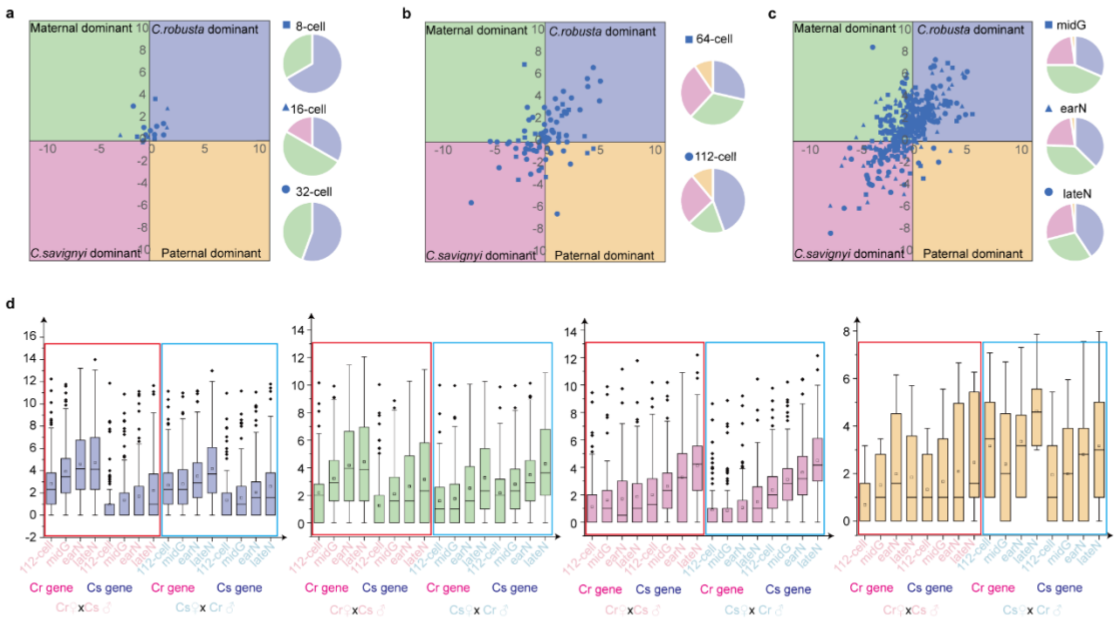

Supplementary Fig. S18 | The allelic gene activation during embryogenesis of hybrid animals. a-c, The quadrant figure was divided into four parts corresponding to four colors. X axis indicated the $\log_2$ transformed expression ratio between genes from *C. robusta* and the allelic genes from *C. savignyi* in Cs♀ × Cr♂ embryos. Y axis indicated the expression ratio between genes from *C. robusta* and the allelic genes from *C. savignyi* in Cr♀ × Cs♂ embryos. Therefore, the first quadrant indicated the level of gene expression from *C. robusta* was higher than its allelic gene from *C. savignyi* in both forward and reverse crosses, then we define it as *C. robusta* dominant. Similarly, the second quadrants indicated gene expression level was maternal dominant in both forward and reverse crosses, the third quadrant was *C. savignyi* dominant in both crosses and the fourth quadrant was paternal dominant in both crosses. Dots of different shapes represented gene expression ratio at different stages. The pie chart showed the proportion of genes in each quadrant. **d**, Expression boxplots from the 112-cell to late-neurula stage. The genes were divided into four groups according to the expression preference.

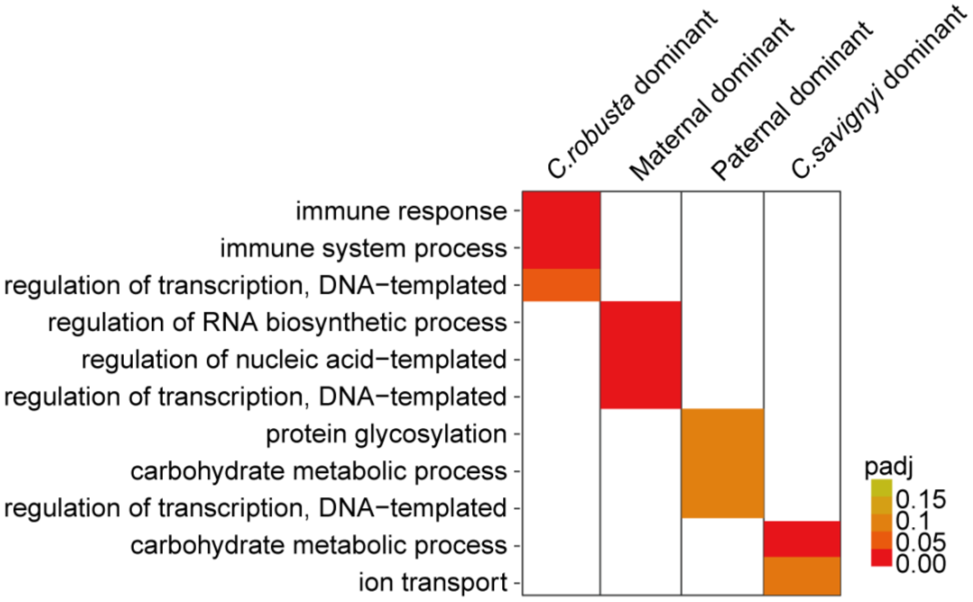

Supplementary Fig. S19 | GO enrichment analysis of genes in different quadrants.

The color indicates the adjust *p*-value.

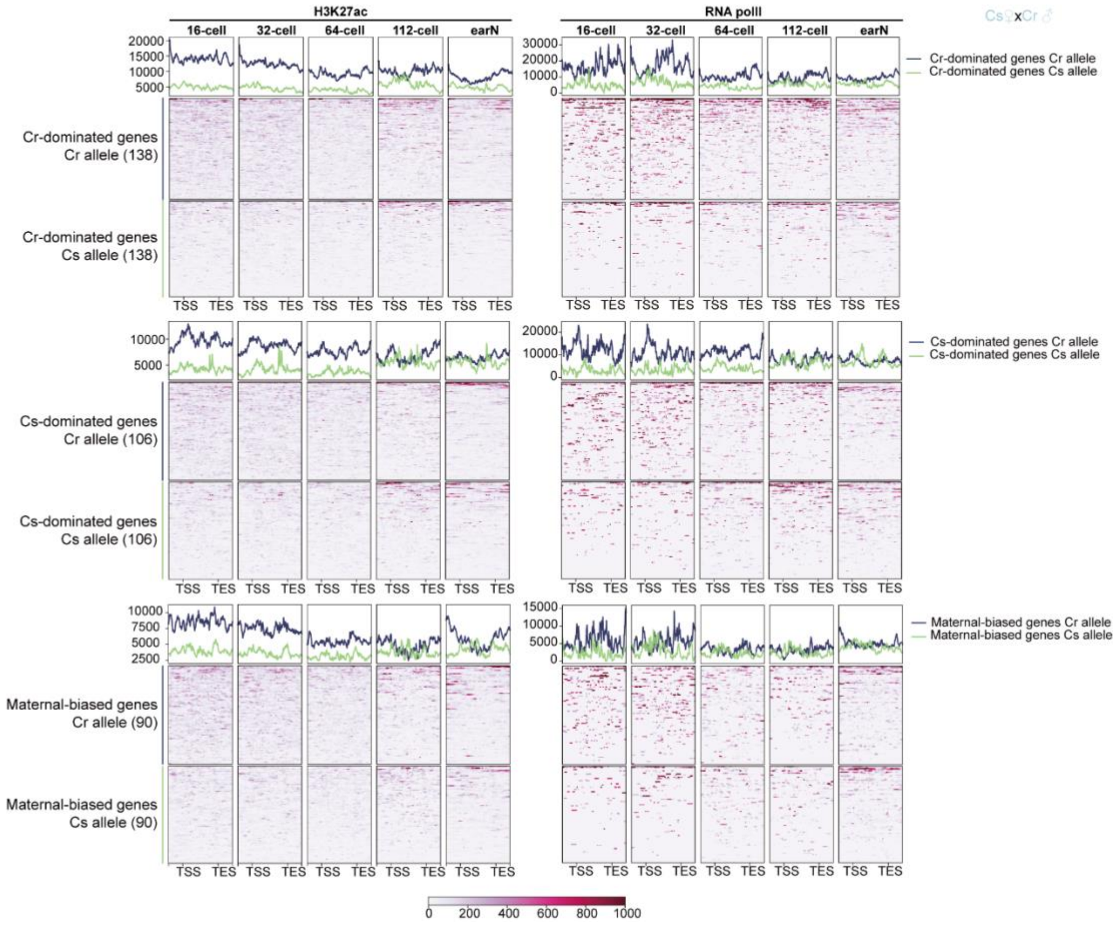

Supplementary Fig. S20 | The CUT&TAG results of H3K27ac and Pol II for biased analysis. Signal profile and heat map of H3K27ac and RNA Pol II CUT&Tag signals across species-biased and parental-biased genes (Cr-dominated genes, Cs-dominated genes, and Maternal-biased genes) in Cs♀ × Cr♂ hybrid embryos. Each line in heat maps shows the normalized signals for a gene (from either *C. robusta* or *C. savignyi* genome) from -800 bp above TSS to +800 bp below TES. While each profile showed by broken line graph is the sum of signals of allele genes in certain genomic location. Broken line profiles in Cr-dominant gene group showed consistence dominance by Cr allele over Cs allele. While in Cs-dominated gene group, Cr allele profile was overtaken by Cs allele profile with the proceeding of developmental stages and activation of genes. Similarly, the maternal-biased gene group showed similar trend with Cs-dominated gene group, which is consistent with the fact that *C. savignyi* as female parent.

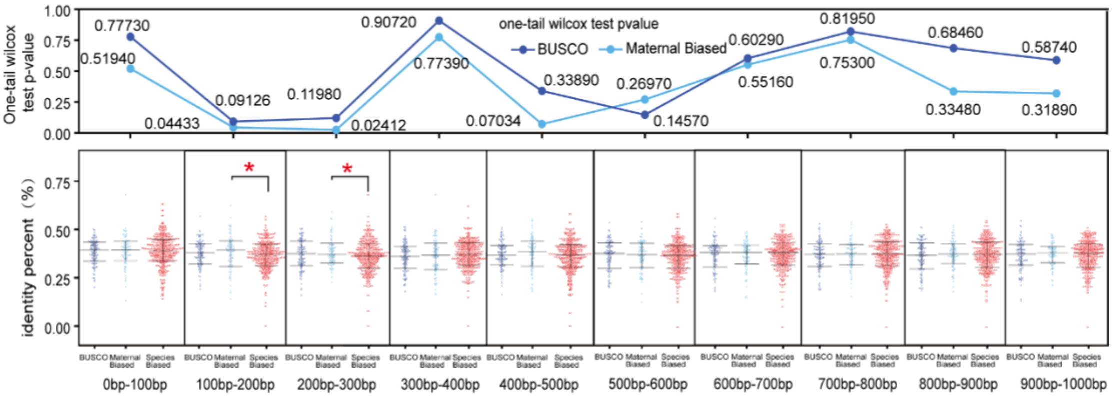

Supplementary Fig. S21 | The sequence identity of 1,000 bp upstream of the initiation codon of CDS in different gene pairs between *C. robusta* and *C. savignyi*. The 1,000 bp upstream sequences were divided every 100 bp, and the gene pairs were divided into species biased, parental biased, and BUSCO genes. The upstream sequences of each gene pair were aligned using BLASTN. The identity percent of each 100 bp were shown. The *p*-value of sequence identity between species-biased group and other groups were labeled.

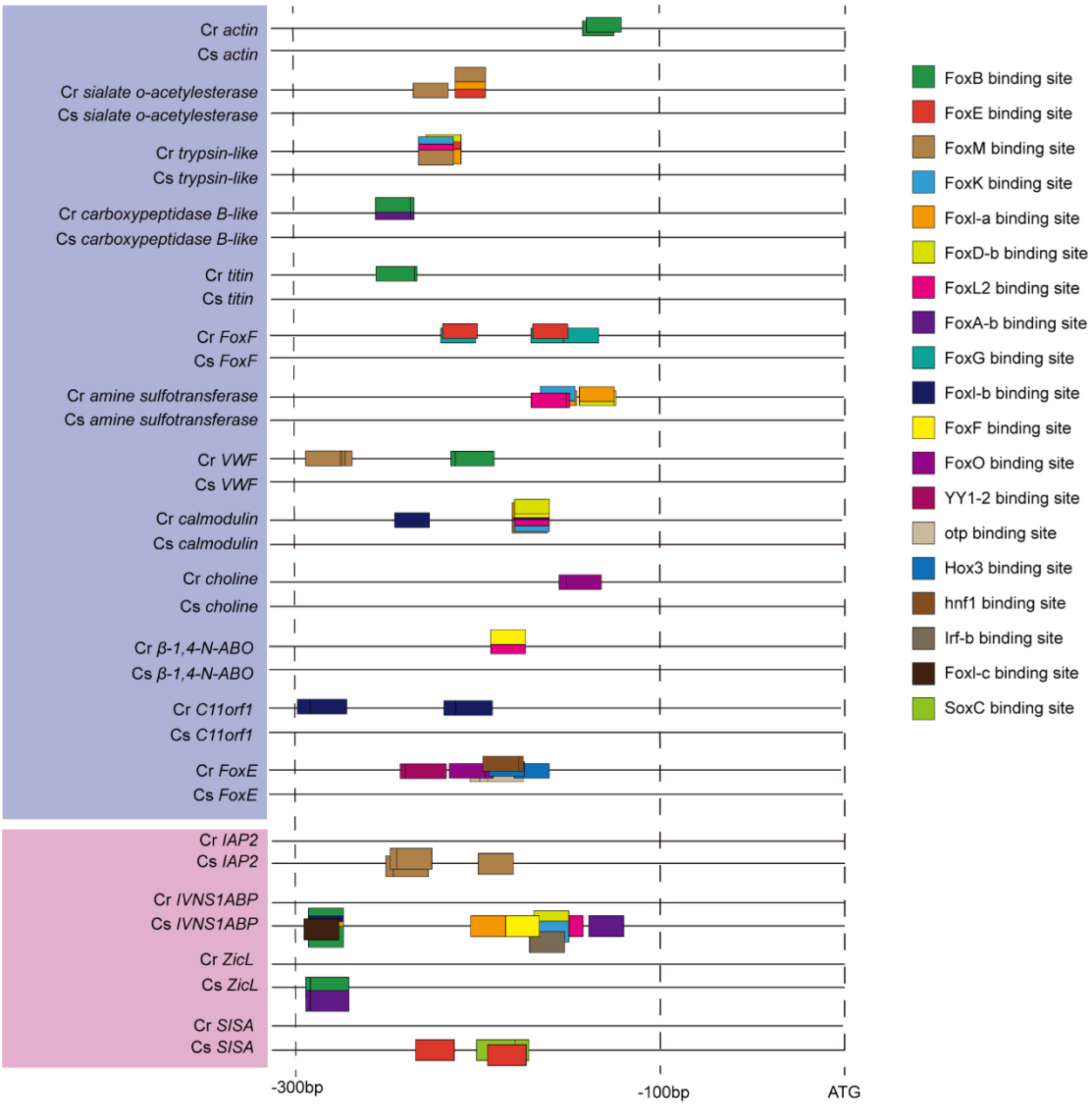

Supplementary Fig. S22 | Motif distribution in species-biased genes from *C. robusta* and *C. savignyi*. The different predicted binding motifs were labeled in different colors.

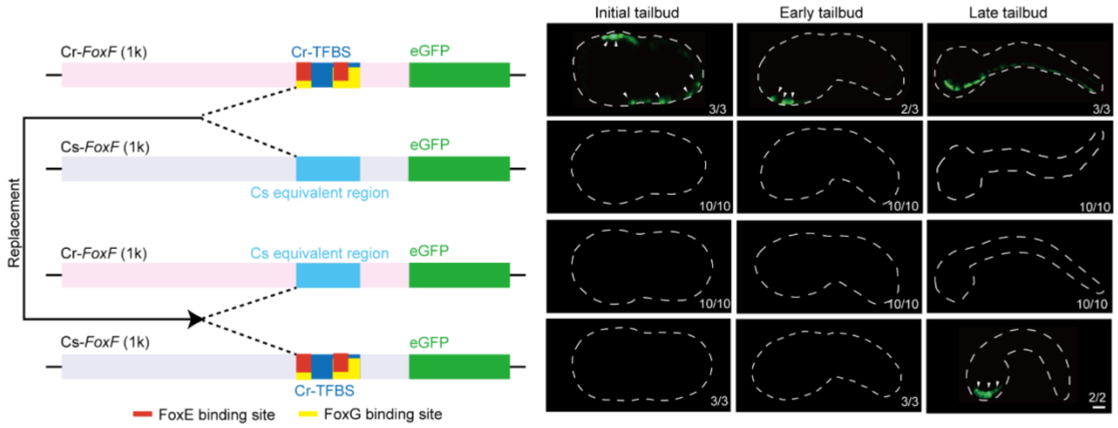

Supplementary Fig. S23 | The promoter activity of *FoxF* from *C. robusta* and *C. savignyi*. The promoters were cloned from *C. robusta* and *C. savignyi*, respectively. The swapped plasmids were also cloned with exchange of motif regions. The plasmids were microinjected into *C. savignyi* embryos, and the GFP signals were observed from early neurula to late tailbud stage. The signals were first presented at initial tailbud stage embryos in *Cr-FoxF* group (white arrowhead). The bar indicates 20 µm.
