# Supplementary material for: Temporospatial hierarchy and allele-specific expression of zygotic genome activation revealed by distant interspecific urochordate hybrids
Source: Nat Commun. 2024 Mar 16;15:2395. doi: 10.1038/s41467-024-46780-0 (PMC10944513; doi:10.1038/s41467-024-46780-0)
Supplement: Supplementary file 3 — Description of Additional Supplementary Files [file 41467_2024_46780_MOESM3_ESM.pdf]

Description of Additional Supplementary Files:

Supplementary Data 1: The statistics of the developmental failure of C. robusta, C. savignyi, and hybrid animals

Supplementary Data 2: The read count ratios of paternal genes to all genes at different developmental stages

Supplementary Data 3: The genes in module 1 and module 2-3 of both directional crosses acquired from WGCNA analysis

Supplementary Data 4: The marker gene list at 64-cell stage of Cr♀ × Cs♂ hybrid Ciona

Supplementary Data 5: The marker gene list at 112-cell stage of Cr♀ × Cs♂ hybrid Ciona

Supplementary Data 6: The marker gene list at early neurula stage of Cr♀ × Cs♂ hybrid Ciona

Supplementary Data 7: The paternal housekeeping gene ratio in each cell from Cr♀ × Cs♂ hybrid

Supplementary Data 8: The marker gene list at 112-cell stage of Cs♀ × Cr♂ hybrid Ciona

Supplementary Data 9: The paternal housekeeping gene ratio in each cell from Cs♀ × Cr♂ hybrid

Supplementary Data 10: The time after the last cell division and cell size in different cell types of C. robusta

Supplementary Data 11: The cell stiffness in different cell linages of C. robusta

Supplementary Data 12: The ratio of siffness related genes in different cell types at 112-cell stage of Cr♀ × Cs♂ hybrid Ciona

Supplementary Data 13: The motif of allelic genes targeting at upstream 100 - 300 bp region from the initiator methionine

Supplementary Data 14: The promoter sequences and swapped sequences guiding EGFP of reporter assay

Supplementary Data 15: The primers used in this study
