# Supplementary material for: Temporospatial hierarchy and allele-specific expression of zygotic genome activation revealed by distant interspecific urochordate hybrids
Source: Nat Commun. 2024 Mar 16;15:2395. doi: 10.1038/s41467-024-46780-0 (PMC10944513; doi:10.1038/s41467-024-46780-0)
Supplement: Supplementary file 5 — Reporting Summary [file 41467_2024_46780_MOESM5_ESM.pdf]

nature portfolio

Corresponding author(s): Bo Dong, Kai Chen, Liang Leng

Last updated by author(s): 2024/02/28

Reporting Summary

Nature Portfolio wishes to improve the reproducibility of the work that we publish. This form provides structure for consistency and transparency in reporting. For further information on Nature Portfolio policies, see our Editorial Policies and the Editorial Policy Checklist.

Statistics

For all statistical analyses, confirm that the following items are present in the figure legend, table legend, main text, or Methods section.

n/a	Confirmed	
☐	☒	The exact sample size (*n*) for each experimental group/condition, given as a discrete number and unit of measurement
☐	☒	A statement on whether measurements were taken from distinct samples or whether the same sample was measured repeatedly
☐	☒	The statistical test(s) used AND whether they are one- or two-sided<br>*Only common tests should be described solely by name; describe more complex techniques in the Methods section.*
☐	☒	A description of all covariates tested
☐	☒	A description of any assumptions or corrections, such as tests of normality and adjustment for multiple comparisons
☐	☒	A full description of the statistical parameters including central tendency (e.g. means) or other basic estimates (e.g. regression coefficient) AND variation (e.g. standard deviation) or associated estimates of uncertainty (e.g. confidence intervals)
☐	☒	For null hypothesis testing, the test statistic (e.g. *F*, *t*, *r*) with confidence intervals, effect sizes, degrees of freedom and *P* value noted<br>*Give P values as exact values whenever suitable.*
☒	☐	For Bayesian analysis, information on the choice of priors and Markov chain Monte Carlo settings
☒	☐	For hierarchical and complex designs, identification of the appropriate level for tests and full reporting of outcomes
☒	☐	Estimates of effect sizes (e.g. Cohen's *d*, Pearson's *r*), indicating how they were calculated

Our web collection on statistics for biologists contains articles on many of the points above.

Software and code

Policy information about availability of computer code

Data collection	no software was used
Data analysis	HISAT2 software v2.0.5, featureCounts (1.5.0-p3), R package WGCNA, Bowtie2 version 2.4.4, bamCoverage version 3.5.1, IGV version2.12.2, DeepTools (v3.5.1), bcl2fastq v1.8.4, Cell Ranger v3.0.2, Seven Bridges platforms pipe line v1.9, Seurat V4

For manuscripts utilizing custom algorithms or software that are central to the research but not yet described in published literature, software must be made available to editors and reviewers. We strongly encourage code deposition in a community repository (e.g. GitHub). See the Nature Portfolio guidelines for submitting code & software for further information.

Data

Policy information about availability of data

All manuscripts must include a data availability statement. This statement should provide the following information, where applicable:

- Accession codes, unique identifiers, or web links for publicly available datasets
- A description of any restrictions on data availability
- For clinical datasets or third party data, please ensure that the statement adheres to our policy

All data generated or analyzed during this study are included in the manuscript and supporting files. The bulk RNA-sequencing, single-cell RNA-sequencing and CUT&Tag sequencing datasets generated in this study have been deposited at NCBI under the accession numbers PRJNA819937, PRJNA820327, PRJNA1003395 and PRJNA1002800. The database used in this study includes JASPAR (https://jaspar.genereg.net/). Source data are provided in the supplementary materials of this

paper.

Research involving human participants, their data, or biological material

Policy information about studies with human participants or human data. See also policy information about sex, gender (identity/presentation), and sexual orientation and race, ethnicity and racism.

Reporting on sex and gender	N/A
Reporting on race, ethnicity, or other socially relevant groupings	N/A
Population characteristics	N/A
Recruitment	N/A
Ethics oversight	N/A

Note that full information on the approval of the study protocol must also be provided in the manuscript.

Field-specific reporting

Please select the one below that is the best fit for your research. If you are not sure, read the appropriate sections before making your selection.

[x] Life sciences [ ] Behavioural & social sciences [ ] Ecological, evolutionary & environmental sciences

For a reference copy of the document with all sections, see nature.com/documents/nr-reporting-summary-flat.pdf

Life sciences study design

All studies must disclose on these points even when the disclosure is negative.

Sample size	The sample sizes were chosen according to the sequence strategy.
Data exclusions	No data were excluded.
Replication	The attempts at replication were successful.
Randomization	The samples were allocated into experimental groups randomly.
Blinding	The investigators were blinded to group allocation during data collection.

Reporting for specific materials, systems and methods

We require information from authors about some types of materials, experimental systems and methods used in many studies. Here, indicate whether each material, system or method listed is relevant to your study. If you are not sure if a list item applies to your research, read the appropriate section before selecting a response.

Materials & experimental systems

n/a	Involved in the study	
[ ]	[x]	Antibodies
[x]	[ ]	Eukaryotic cell lines
[x]	[ ]	Palaeontology and archaeology
[ ]	[x]	Animals and other organisms
[x]	[ ]	Clinical data
[x]	[ ]	Dual use research of concern
[x]	[ ]	Plants

Methods

n/a	Involved in the study	
[x]	[ ]	ChIP-seq
[x]	[ ]	Flow cytometry
[x]	[ ]	MRI-based neuroimaging

Antibodies

Antibodies used	antibody: Acetyl-Histone H3-K27 Rabbit pAb, supplier name: abclonal, catalog number: A7253, clone name: none, lot number: 5500016907; antibody: RNA pol II antibody (mAb), supplier name: Active motif, catalog number: 39497, clone name: 4H8, lot number: 8322027;
Validation	antibody: Acetyl-Histone H3-K27 Rabbit pAb, species: Human, Mouse, Rat, Other (Wide Range), application: WB/IHC-P/IF/ICC/IP/

ChIP/ChIP-seq, website: https://abclonal.com.cn/catalog/A7253; antibody: RNA pol II antibody (mAb), species: Budding Yeast, C. elegans, Human, Mouse, Rat, application: WB/IF/ICC/ChIP/ChIP-seq, website: https://www.activemotif.com/catalog/details/39097/rna-pol-ii-antibody-mab;

Animals and other research organisms

Policy information about studies involving animals; ARRIVE guidelines recommended for reporting animal research, and Sex and Gender in Research

Laboratory animals	The study did not involve laboratory animals.
Wild animals	Adult ascidians (Ciona robusta and Ciona savignyi) were collected in Weihai, China, and transported and bred in seawater at 18°C in the laboratory. The animals were dead after sperms and eggs extraction.
Reporting on sex	The sex of the embryos is unknown
Field-collected samples	No field collected samples were used in the study.
Ethics oversight	As invertebrate organisms, no ethical permission was required

Note that full information on the approval of the study protocol must also be provided in the manuscript.

Plants

Seed stocks	*Report on the source of all seed stocks or other plant material used. If applicable, state the seed stock centre and catalogue number. If plant specimens were collected from the field, describe the collection location, date and sampling procedures.*
Novel plant genotypes	*Describe the methods by which all novel plant genotypes were produced. This includes those generated by transgenic approaches, gene editing, chemical/radiation-based mutagenesis and hybridization. For transgenic lines, describe the transformation method, the number of independent lines analyzed and the generation upon which experiments were performed. For gene-edited lines, describe the editor used, the endogenous sequence targeted for editing, the targeting guide RNA sequence (if applicable) and how the editor was applied.*
Authentication	*Describe any authentication procedures for each seed stock used or novel genotype generated. Describe any experiments used to assess the effect of a mutation and, where applicable, how potential secondary effects (e.g. second site T-DNA insertions, mosiacism, off-target gene editing) were examined.*
